# Au Nanoparticles@Si Nanowire Oligomer Arrays for SERS: Dimers Are Best

**DOI:** 10.1021/acsami.4c10004

**Published:** 2024-07-26

**Authors:** Theresa Bartschmid, Johannes Menath, Lukas Roemling, Nicolas Vogel, Furkan Atalay, Amin Farhadi, Gilles R. Bourret

**Affiliations:** † Department of Chemistry and Physics of Materials, University of Salzburg, Jakob Haringer Strasse 2A, A-5020 Salzburg, Austria; ‡ Institute of Particle Technology, Friedrich-Alexander University Erlangen-Nürnberg, Cauerstrasse 4, 91058 Erlangen, Germany

**Keywords:** silicon nanowires, dimers, Au nanoparticles, metal-assisted chemical etching, colloidal lithography, SERS

## Abstract

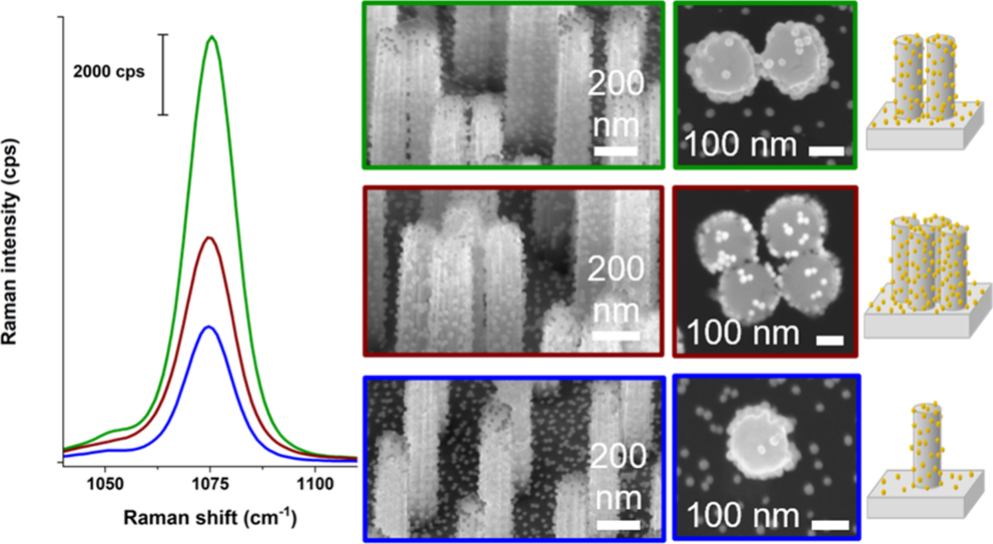

We report the synthesis of vertically aligned silicon nanowire (VA-SiNW) oligomer arrays coated with Au nanoparticle (NP) monolayers via a combination of colloidal lithography, metal-assisted chemical etching, and directed NP assembly. Arrays of SiNW monomers (i.e., isolated NWs), dimers, and tetramers are synthesized, decorated with AuNPs, and tested for their performance in surface-enhanced Raman spectroscopy. The ∼20 nm AuNPs easily enter within the ca. 40 nm gaps of the SiNW oligomers, thus reaching the hot spot region. At 785 nm excitation, the AuNPs@SiNW dimer arrays provide the highest Raman signal, in agreement with electromagnetic simulations showing a high electric field enhancement at the Au/Si interface within the dimer gap region.

## Introduction

Under light irradiation, plasmonic metal nanostructures can efficiently enhance the incident electric field (E-field) in the near-field region.^1−5^ This resonance effect has long been used for sensing applications based on surface-enhanced Raman scattering (SERS), where the increase of both the incident and scattered light intensities at the metal surface can lead to dramatic enhancement of the Raman signal.^2−4,6,7^ The SERS effect is highest within electromagnetic hot spots, which can be efficiently generated between metal nanoparticle (NP) dimers that are separated by nanometer scale gaps.^2−4,8−13^ Alternatively, the combination of plasmonic and resonant dielectric nanostructures has emerged as an efficient strategy to enhance the E-field at the metal/dielectric interface without requiring nanoscale gaps,^7,14^ which can be challenging to fabricate with the required precision.^7,14−20^ For example, vertically aligned silicon nanowire (VA-SiNW) arrays can provide moderate E-field enhancements at most visible wavelengths. They can sustain Mie and Fabry-Pérot resonances, as well as leaky waveguided modes, which are all highly dependent on the VA-SiNW array geometry, i.e., NW diameter and length, and array pitch.^7,15,18,21−31^ When coated with a layer of AuNPs, VA-SiNW arrays show an increase in SERS intensity by almost 1 order of magnitude when compared to a similar AuNP layer on a flat Si substrate.^7^ This significant increase is due to the enhanced E-field generated at the Au/Si interface and the three-dimensional geometry of the SiNW array that provides a high density of binding sites for the analyte molecules, which is probed by the micrometer-sized Raman excitation spot.

A further enhancement of the E-field has been shown when two of such pillars synthesized by electron-beam lithography are brought in close proximity,^21−23,25,26^ in analogy to the plasmonic hotspots that form within nanoscale metallic gaps.^8,10,21,32^ Due to their small length, these dimers only supported Mie resonances. Previous simulations suggested that a similar near-field coupling is also expected between the guided modes of micron-long VA-SiNWs arranged in a dimer configuration.^26^ Combined with plasmonic NP monolayers, such VA-SiNW dimer arrays could provide maximum E-field enhancement, a high surface area, and a large three-dimensional hot spot density. Because the Raman excitation laser spot size is commensurate with the length of SiNWs, AuNPs@SiNW dimer arrays should provide even higher Raman signals. Additionally, combining AuNPs@SiNWs into oligomers, such as tetramers, might increase further the E-field enhancement and thus the Raman signal. To date, such AuNPs@SiNW oligomer arrays have not been synthesized, optically characterized, or tested for SERS.

VA-SiNW arrays can be synthesized via bottom-up methods such as vapor–liquid–solid (VLS) synthesis,^33,34^ top-down etching syntheses such as (deep) reactive-ion etching (D)RIE,^35−38^ or metal-assisted chemical etching (MACE).^31,39^ MACE is a versatile and high-throughput wet chemical etching technique that can be used to synthesize a variety of nanostructured silicon substrates.^30,31,39^ It is based on the preferential reduction of a strong oxidant, usually hydrogen peroxide (H_2_O_2_), at the surface of a catalytic etching mask, often made of Au. In the presence of hydrofluoric acid (HF), this active mask etches Si in a highly anisotropic fashion. MACE can successfully transfer a variety of metal nanostructure patterns into the silicon substrate and is often used to synthesize VA-SiNW arrays using metal holey meshes perforated with circular holes.^7,15,30,31,39,40^ MACE does not require expensive lab equipment, is cost-effective, relatively fast, and can be used with different catalyst composition and geometry, and substrate materials.^30,31,39,41^ Additionally, MACE is especially effective at preparing high-aspect-ratio Si nanostructures with smooth walls, and can be CMOS-compatible using appropriate etching masks.^42−46^

Instead of using expensive lithography techniques that rely on photo- or electron-beam lithography to pattern the catalytic mask, colloidal lithography, which is based on the self-assembly of polymer spheres into a well-ordered monolayer, has appeared as an affordable, parallel, and large-scale approach to pattern substrates with subwavelength resolution.^47,48^ To date, colloidal lithography has been combined with MACE to synthesize hexagonal arrays of VA-SiNWs with circular,^31^ elliptic,^49^ or hexagonal^50^ cross sections. Binary VA-SiNW arrays composed of SiNWs with two distinct diameters can also be synthesized by assembling particles of different sizes during colloidal lithography,^51^ while diameter gradients can be encoded either at the macroscale within the VA-SiNW array or at the single nanowire level by using sequential MACE and KOH etching steps.^30^ Additionally, the metal film located at the bottom of the SiNW array after MACE can also be used to pattern the nanowires with a variety of conducting and insulating materials.^14,15,52^ Thus, the combination of colloidal lithography and MACE is highly versatile in creating silicon nanostructures.

Recently, soft, core–shell colloidal particles have been shown to form a variety of particle dimer, trimer, tetramer, and oligomer structures when deposited onto a solid substrate.^53^ Here, we take advantage of these colloidal monolayers with an unconventional arrangement as templates for preparing the catalytic MACE masks. After MACE, the colloidal template geometry is transferred into the silicon substrate to synthesize arrays of VA-SiNW dimers and tetramers with gap sizes of ca. 40 and 60 nm, respectively, uniformly over macroscopic areas. Functionalization of the Si surface with positively charged amino groups is used to form a dense monolayer of negatively charged AuNPs at the SiNW surface via electrostatic attraction, as previously reported by our group.^7^ Raman investigations show that the AuNPs@SiNW dimer arrays provide a SERS signal higher than that of the AuNPs@SiNW monomer arrays and the AuNPs@SiNW tetramer arrays. This demonstrates that the SiNW dimer morphology provides the largest E-field enhancement at 785 nm, which is confirmed by finite-difference time-domain (FDTD) electromagnetic simulations.^7,54,55^

## Results and Discussion

**Figure 1:**
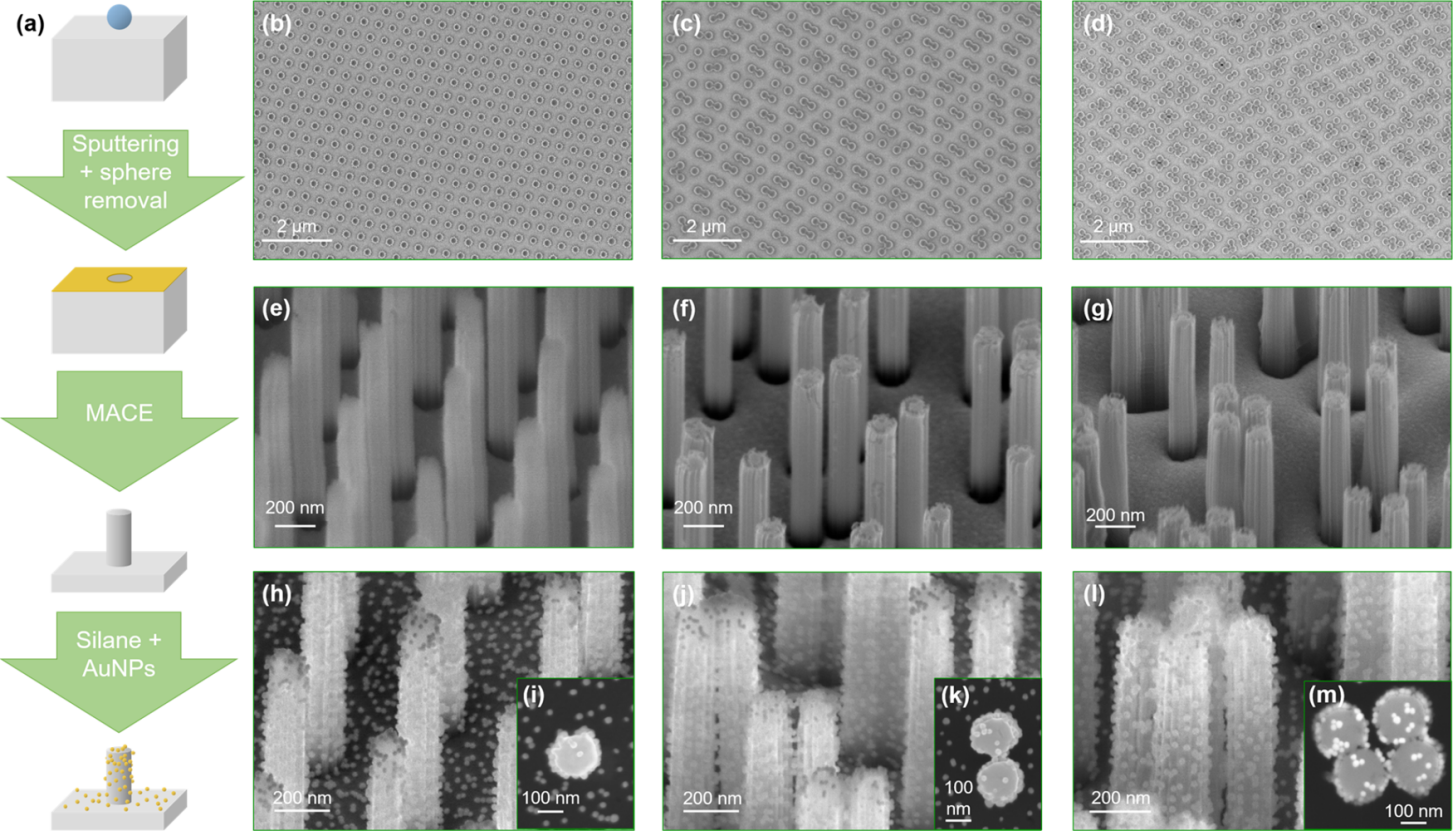
Overview of the synthesis procedure of SiNW monomer, SiNW dimer, and SiNW tetramer arrays including colloidal lithography, sputtering, MACE, surface functionalization with an aminosilane and incubation in AuNP solution (a), with scanning electron microscopy (SEM) images corresponding to the monomer (b, e, h, i), dimer (c, f, j, k), and tetramer samples (d, g, l, m) at different stages of the synthesis.

VA-SiNW arrays were prepared via colloidal lithography using core–shell SiO_2_@PDMAEMA (poly(2-(dimethylamino)ethyl methacrylate)) colloidal particles with a SiO_2_ diameter of (170 ± 7) nm and hydrodynamic diameter of the core–shell particle of (481 ± 75) nm (Figure 1).^53^ These particles were self-assembled at the air–water interface and compressed using a Langmuir trough. After transfer of this interfacial monolayer with increasing compression, anisotropic colloidal arrangements with an increased fraction of dimeric and tetrameric assemblies were formed on the substrate (Figure 1b–d). After size reduction and the deposition of a thin metal film, anisotropic Au nanohole arrays were formed and used as etching masks in MACE (Figure 1a).^7,15,30,31,53,56^ During MACE, the anisotropic and preferential dissolution of the silicon in direct contact with the Au film produces an array of well-defined VA-SiNWs, which preserve the original metal film morphology (Figure 1e–g). After functionalization with (3-aminopropyl)triethoxysilane (APTES), the nanostructured Si substrates with positively charged surface groups were incubated in a solution of negatively charged, citrate-stabilized AuNPs, prepared via a modified Turkevich synthesis.^7,57^ A dense monolayer of randomly arranged AuNPs formed on all of the SiNW samples prepared in this work (Figure 1h–m), similarly to our previous results obtained on isolated SiNWs,^7^ which demonstrates the robustness and reliability of this assembly approach.

To investigate the SERS performance of these hybrid surfaces, MACE was performed on a “gradient” sample, where an interfacial monolayer of the SiO_2_–PDMAEMA particles was transferred under continuous compression while simultaneously removing the Si slide from the Langmuir trough.^53^ After plasma treatment to remove the organic shell, sputtering of aluminum-doped zinc oxide (AZO) and Au, and subsequent removal of the templating SiO_2_ colloidal particles, MACE produced a gradient of nanostructured silicon with defined regions of ca. 5 × 14 mm^2^ composed of (isolated) SiNW monomer arrays, SiNW dimer arrays and SiNW tetramer arrays, which were identified and selected via SEM (Figure 1e–g). While the SiNW monomer sample is exclusively composed of SiNW monomers, ca. 61% of the SiNWs are present in a dimer configuration on the dimer sample, and 37% of SiNWs were arranged in a tetramer configuration on the tetramer sample. The average gap size of the SiNW dimer and tetramer arrays was 38 ± 21 and 60 ± 20 nm, respectively. Remarkably, the ca. 17 nm small AuNPs are seemingly able to efficiently reach the dimer and tetramer gap region (see Figure 1k,m). A collection of SEM images recorded on different regions of the AuNPs@SiNW monomer, dimer, and tetramer arrays can be found in Figure S1, which illustrates the homogeneity of the SiNW structures and their coverage with AuNPs over large areas.

### Optical Properties

**Figure 2:**
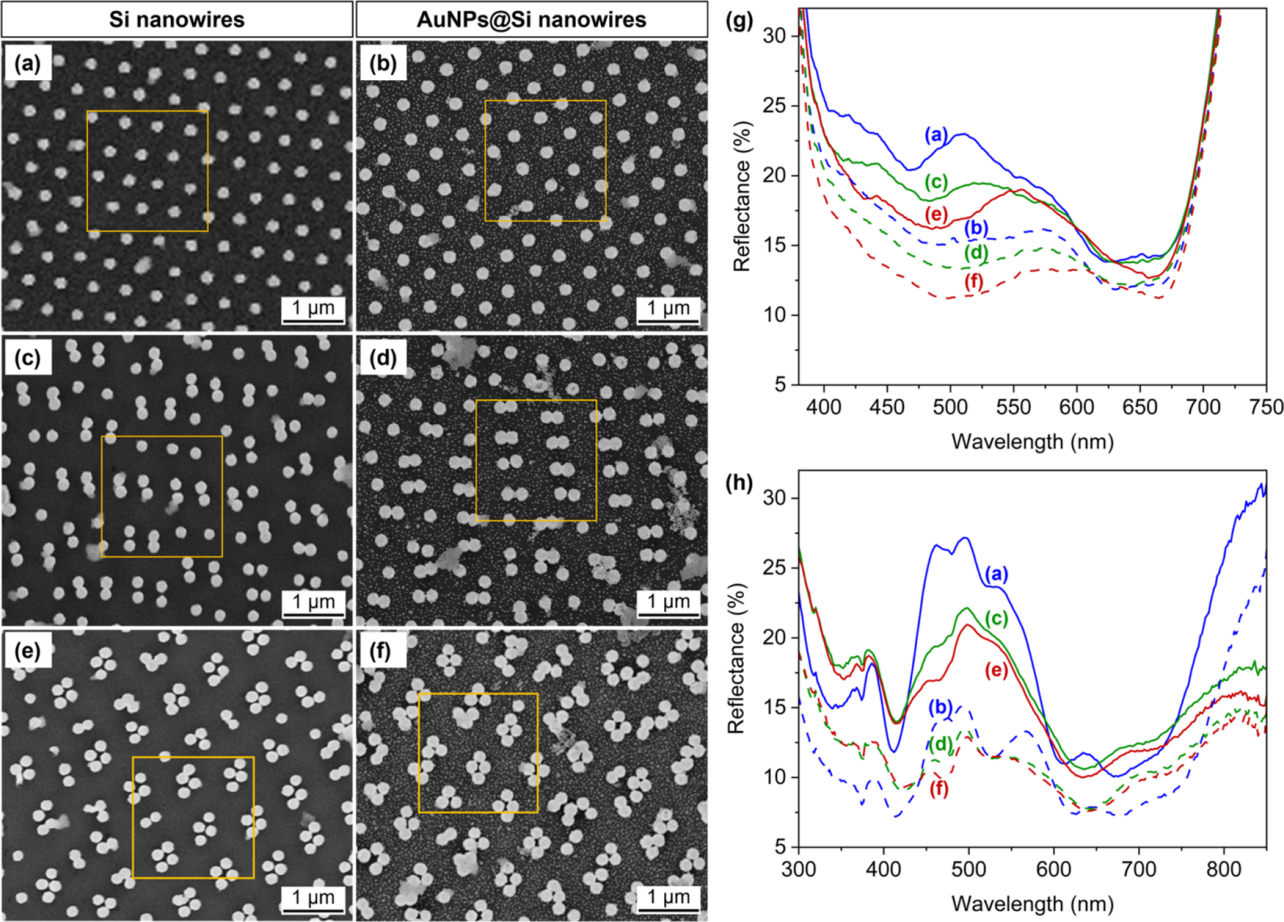
Correlative SEM UV–vis measurements on 2 × 2 μm^2^ regions indicated by orange squares on SEM images (a–f). (a, b) SiNW monomer arrays without (a) and with AuNPs (b). (c, d) SiNW dimer arrays without (c) and with AuNPs (d). (e, f) SiNW tetramer arrays without (e) and with AuNPs (f). (g) Corresponding microspectroscopic UV–vis spectra, acquired in the orange squares shown in (a–f). Blue lines: SiNW monomer arrays without AuNPs (solid line) and with AuNPs (dotted line). Green lines: SiNW dimer arrays without AuNPs (solid line) and with AuNPs (dotted line). Red lines: SiNW tetramer arrays without AuNPs (solid line) and with AuNPs (dotted line). (h) Macroscopic UV–vis spectra extending to the >700 nm range, and acquired in a region of ca. 7 mm^2^ on the same samples (a–f) are shown for comparison. Same color legend as that for (g).

The synthesized SiNW and AuNPs@SiNW arrays were studied via correlated UV–vis microspectroscopy and SEM (Figure 2). A 2 × 2 μm^2^ sample area was determined via SEM before recording the corresponding reflectance spectra using the dedicated aperture of a homemade optical microscope coupled with a UV–vis spectrometer (more details in the experimental section in the SI). The exact sample location used to acquire each UV–vis spectrum is indicated by the orange squares in Figure 2a–f and corresponds to ca. 15–25 SiNWs. Ensemble reflectance spectra performed on millimeter-sized areas are shown in Figure 2h for comparison.

As seen in Figure 2g, the reflectance minimum at around 470 nm, corresponding to the excitation of the SiNW HE_12_ waveguiding mode, is slightly red-shifted to ca. 480 nm when the SiNWs form dimers and to ca. 485 nm for the SiNW tetramers.^21,26,29,30^ The HE_11_ waveguiding mode around 650 nm shows a similar trend.^21,26,29,30^ Taken together, these results suggest that near-field coupling occurs within the dimer and tetramer samples.^26^ The addition of AuNPs leads to a reduced reflectance for all three types of substrates, which can be attributed to increased absorption within the AuNPs due to LSPR excitation and inter/intraband transitions, and to the reduction of the reflective Si surface area by the presence of AuNPs.^3,4,7^

Microspectroscopic UV–vis spectra probing a 2 × 2 μm^2^ region were recorded in the ca. 380–720 nm range. Since the SERS measurements were performed at 785 nm, macroscopic UV–vis spectra were acquired up to 850 nm on ca. 7 mm^2^ areas (see Figure 2h). Overall, both types of UV–vis measurements show similar features, with two reflectance dips corresponding to guided modes and a decrease in reflectance observed after the addition of AuNPs. The spectral shift of the waveguiding modes on the dimer and tetramer samples is not as well defined on the macroscopic UV–vis spectra, which we attribute to the sample geometrical inhomogeneities. Two important observations can be made from these macroscale UV–vis spectra: (i) The guided modes are partially excited at 785 nm on all samples, i.e., 785 nm is neither on-resonance nor completely off-resonance with the HE_11_ mode. (ii) The AuNPs@SiNW monomer samples have a larger reflectance than the dimer and tetramer samples at 785 nm, which could be attributed to an overall lower interaction with light at this wavelength, potentially leading to a lower E-field enhancement, regardless of the origin of the field enhancement.

### Raman Measurements

**Figure 3:**
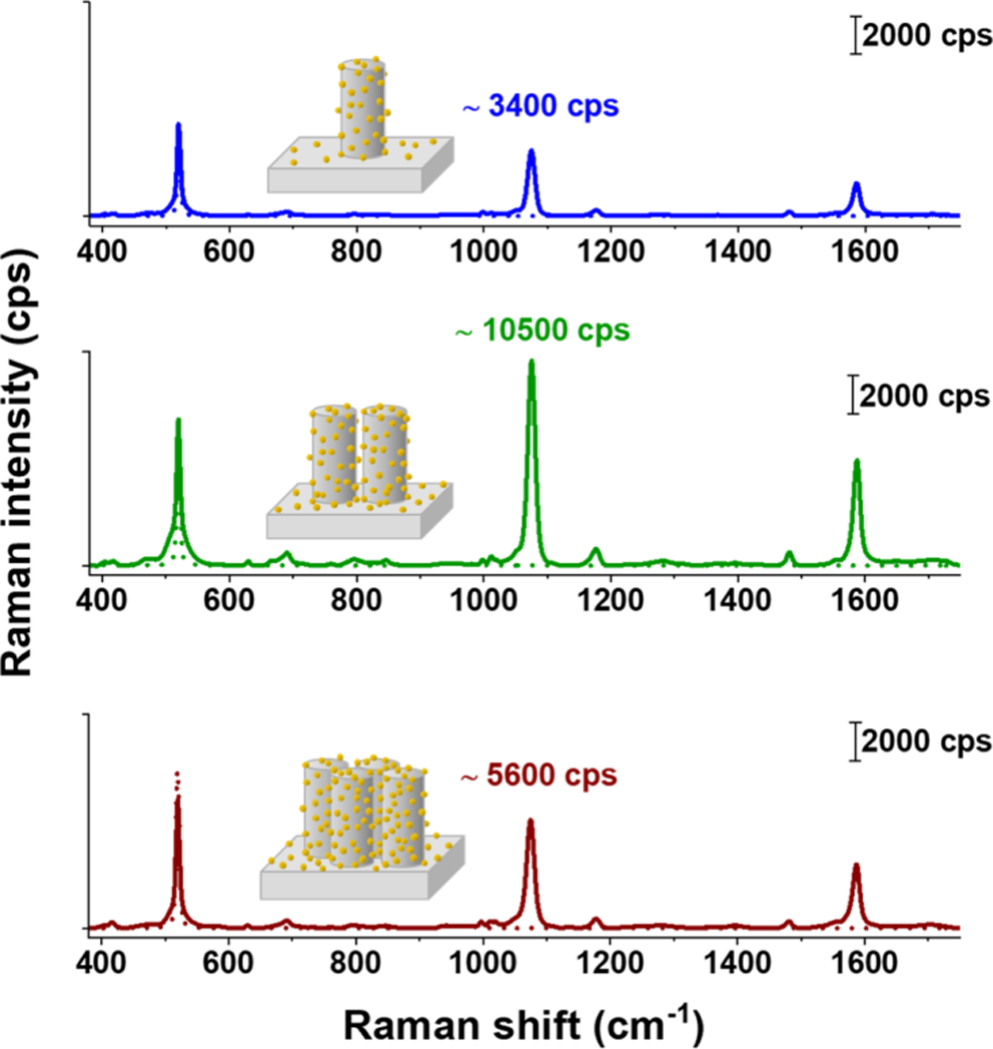
Baseline corrected Raman spectra recorded with 785 nm laser excitation on the SiNW monomer arrays with and without AuNPs (blue solid and dashed lines), on the SiNW dimer arrays with and without AuNPs (green solid and dashed lines), and on the SiNW tetramer arrays with and without AuNPs (red solid and dashed lines) together with the respective mean values for the peak of the analyte 4-MBA at 1075 cm^–1^.^58^

SiNW monomer arrays, SiNW dimer arrays, and SiNW tetramer arrays with and without AuNPs were investigated with a confocal Raman microscope. The analyte molecule 4-mercaptobenzoic acid (4-MBA) was drop-cast on the substrates. A depolarized laser at an excitation wavelength of 785 nm was used with a 10 mW power and a 10× objective with a numerical aperture (NA) of 0.25, leading to a spot size of approximately 3.1 μm in the substrate plane. Details regarding the sample preparation can be found in the experimental section in the SI. Figure 3 shows averaged Raman spectra of analyte molecule 4-MBA on the different substrates. The peaks at 1588 and 1075 cm^–1^ can be assigned to vibrations of the 4-MBA aromatic ring.^58^ No signal from the analyte was detected on the bare SiNW samples (i.e., without AuNPs, see dotted line spectra in Figure 3), while a large Raman signal was measured on all of the AuNPs@SiNW array substrates. The AuNPs@SiNW dimer substrates showed approximately three and two times higher signal intensities compared to the AuNP@SiNW monomer and the AuNP@SiNW tetramer substrates, respectively, thus indicating that the dimer geometry leads to a higher SERS effect at 785 nm.

All types of samples showed a slightly different SiNW density, with ca. 4 SiNWs/μm^2^ for the isolated SiNW monomer arrays, 5 SiNWs/μm^2^ for the SiNW dimer arrays, and 6 SiNWs/μm^2^ for the SiNW tetramer arrays. Thus, the total number of AuNPs present in the illumination regions cannot account by itself for the increase in the Raman signal measured on the dimer samples. Additionally, since the three substrates were cut from the same (gradient) sample, they were prepared via MACE, functionalized with amino groups, and coated with AuNPs under identical conditions and should have similar AuNP densities at the Si surface. The relative standard deviations (RSDs) of the Raman signal measured on the AuNPs@SiNW monomer, dimer, and tetramer arrays are 35% (which is on par with previously reported data for similar structures),^7^ 43%, and 20%, respectively. Overall, purely structural aspects cannot account for the increased SERS intensity of the dimer samples.

We next assessed the role of the target molecule and demonstrated the potential use of these Au–Si hybrid structures as SERS substrates by performing Raman measurements with 785 nm laser excitation for malachite green (MG) and Rhodamine 6G (R6G) (Figure S2). A high Raman signal was measured on all substrates, and a similar trend in the Raman enhancement was observed, i.e., dimers > tetramers > monomers.

When the laser excitation wavelength was changed to 532 nm for the Raman measurements of 4-MBA, however, no difference between the three different substrates was observed (Figure S3). The Raman signal intensities are considerably lower than those measured at 785 nm on all types of substrates. In this case, the AuNPs@SiNW dimer arrays do not overperform, which hints that an increased near-field effect at 785 nm (or increased light-matter interaction more generally) rather than structural effects (i.e., varying amounts of AuNPs, longer SiNWs, etc.) causes the larger SERS signal measured on the dimer sample.

### Electromagnetic FDTD Simulations

**Figure 4:**
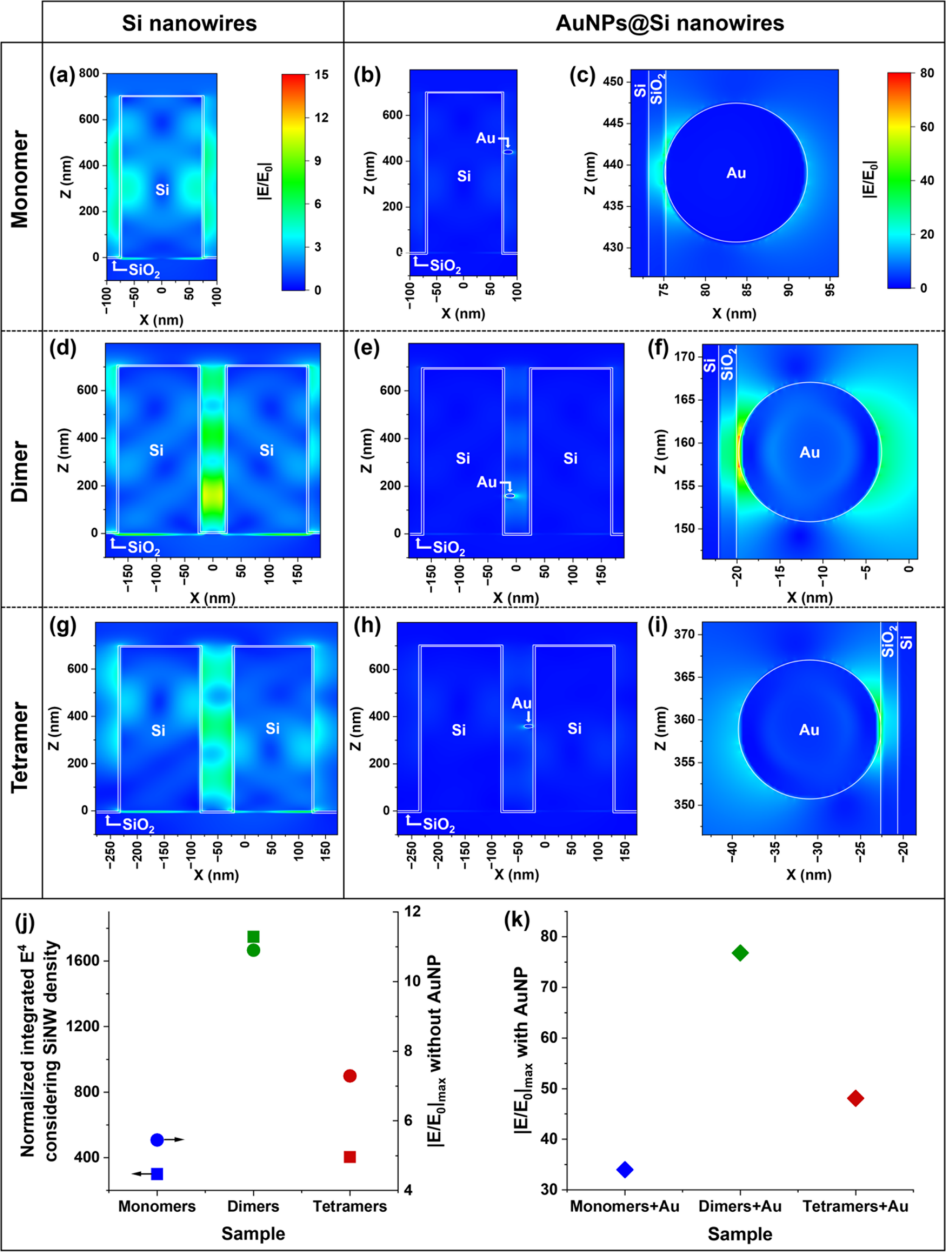
(a, d, g) Simulated E-field enhancement maps of a Si nanowire monomer (a), dimer with a 40 nm gap (d), and tetramer with a 60 nm gap (g), on the same color scale, shown in (a). Corresponding E-field maps with a AuNP (17 nm diameter) located in a region of increased E-field for the monomer (b, c), dimer (e, f), and tetramer (h, i) using the same color scale, shown in (c). A native 2-nm-thick SiO_2_ layer is used in all of the simulations. (j) *E*^4^ integrated over a 5 nm shell around the SiNW geometry, normalized by the integration volume, and multiplied by the SiNW density (square symbols, left axis), and maximum enhancement of the E-field strength without AuNP (full circle symbols, right axis). (k) Maximum enhancement of the E-field strength with a AuNP located in the region of increased field intensity for the three different SiNW samples. Light was injected along the *Z* axis and polarized along the *X* axis with a 785 nm wavelength.

Electromagnetic simulations using the FDTD method (Figure 4) were performed to rationalize the enhanced Raman signal measured on the AuNPs@SiNW dimer arrays with a 785 nm excitation. The enhancement of the E-field strength was simulated on an idealized SiNW monomer array (Figure 4a), a SiNW dimer array (Figure 4d), and a SiNW tetramer array (Figure 4g), along with their corresponding AuNPs@SiNW hybrid structures, where one AuNP was located in the region of highest E-field (Figure 4b,c,e,f,h,i, respectively), i.e., in the gap region for the dimers and tetramers. Figure S4 in the Supporting Information shows the E-field monitors for each array geometry and the distance used to determine the average gap size in the SiNW tetramers. The simulated maximum enhancement of the E-field amplitude is ca. twice as high for the SiNW dimer than for the SiNW monomer array and 1.5 times higher compared to the SiNW tetramer (Figure 4j). In the dimer and tetramer configuration, the E-field is confined within the gap region due to the near-field coupling of the various modes excited in the SiNWs.^22,26,59^ When a single AuNP is added to this region of highest E-field, the maximum E-field is enhanced by a factor of ca. 6.2, 7.0, and 6.6 for the AuNPs@SiNW monomer array, the AuNPs@SiNW dimer array, and the AuNPs@SiNW tetramer array, respectively (Figure 4j,k). In agreement with previous works on similar structures,^7,18^ the E-field is most enhanced at the Au/Si interface.

In summary, these simulations show that the AuNPs@SiNW dimer array provides a maximum E-field enhancement that is ca. 2.3 times larger than that on the AuNPs@SiNW monomer array and ca. 1.6 times larger than that on the AuNPs@SiNW tetramer array (see Figure 4k). Since the Raman intensity scales with the fourth power of the E-field amplitude,^2,4^ i.e., *E*^4^, we would expect the AuNPs@SiNW dimer array to provide a Raman signal that is 28 times higher than the one measured on the AuNPs@SiNW monomer array and ca. 7 times higher compared to the AuNPs@SiNW tetramer array if only one molecule was sitting in the region of highest E-field. However, because these are three-dimensional substrates, the Raman signal results from the light scattered by all of the analyte molecules and not just the one located in the “hottest” spot. Thus, comparing the *E*^4^ enhancement averaged over the entire SiNW surface should provide a more accurate depiction of the SERS experiments where the Raman laser spot probes the entire SiNW length and all of the analyte@AuNPs it carries. To avoid running prohibitively demanding simulations by saturating the SiNW surface with AuNPs, we integrated the *E*^4^ enhancement in a 5 nm shell around the simulated SiNWs (i.e., with no AuNP present), normalized to the integration volume and multiplied by the SiNW density of the respective sample (Figure 4j). The resulting averaged and normalized *E*^4^ enhancement provided by the SiNW dimers is ca. 5.8 times larger than the one provided by SiNW monomers and 4.4 times larger than the SiNW tetramers (Figure 4j), which qualitatively agrees with our experimental SERS results, where the AuNPs@SiNW dimer configuration gave a Raman signal that is 3 and 2 times higher than those of the AuNPs@SiNW monomers and the AuNPs@SiNW tetramers, respectively. Additionally, FDTD simulations performed at 532 nm show that the SiNW oligomers provide a much lower enhancement of both the maximum E-field and integrated *E*^4^ (Figure S5) at 532 nm, in agreement with the lower SERS signal measured at 532 nm in the experiment (Figure S3).

**Figure 5:**
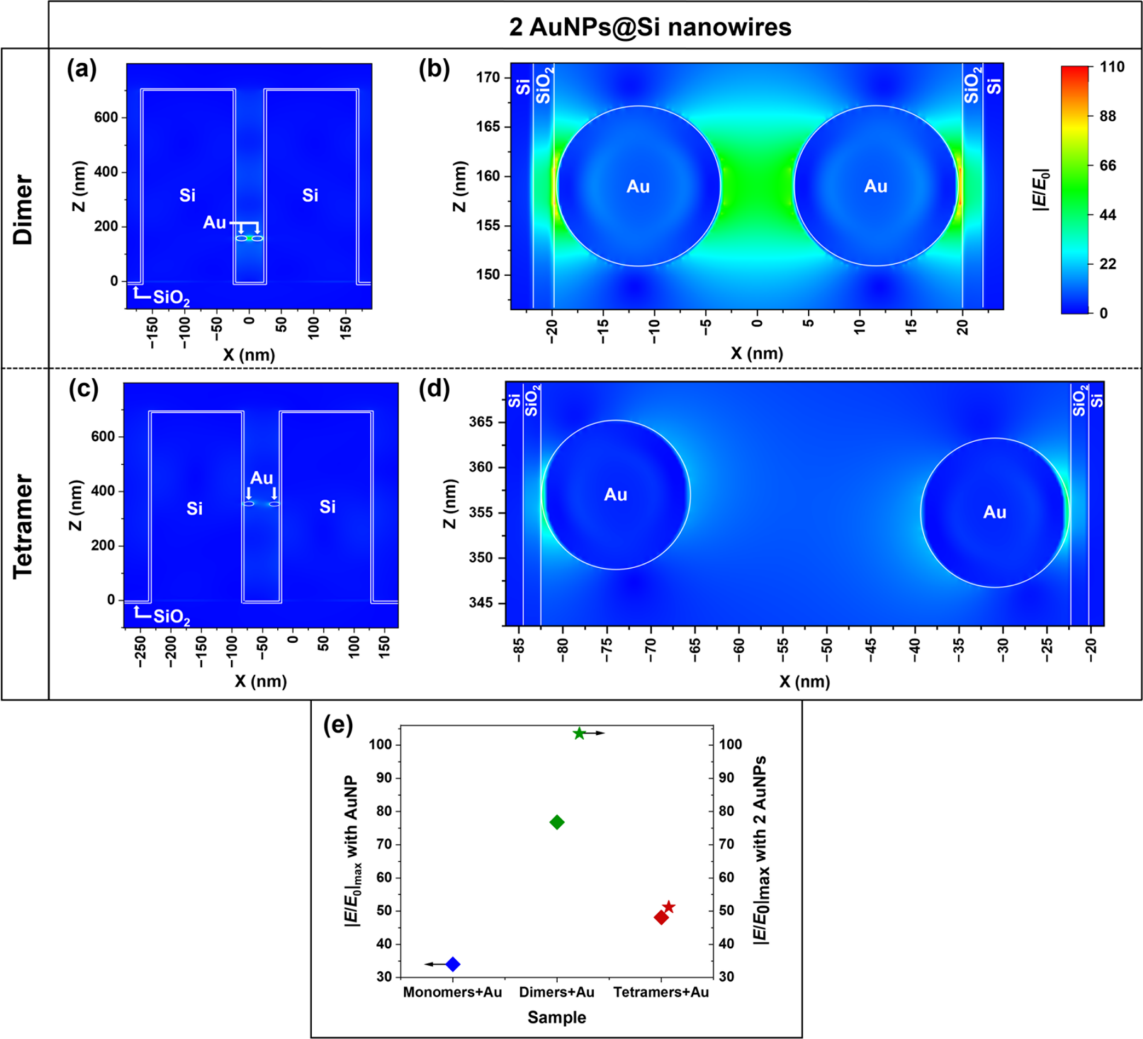
Simulated E-field enhancement maps of a SiNW dimer with a 40 nm gap with two AuNPs located in the dimer gap in regions of increased E-field (a, b) and tetramer with a 60 nm gap with two AuNPs located in the tetramer gap in regions of increased E-field (c, d), same color scale, shown in (b). AuNPs have a diameter of 17 nm. A native 2-nm-thick SiO_2_ layer is used in all of the simulations. (e) Maximum enhancement of the E-field strength with a AuNP located in the region of increased field intensity for the three different SiNW samples (left) and with two AuNPs in the gap regions of SiNW dimer and tetramer arrays (right). Light was injected along the *Z* axis and polarized along the *X* axis with a 785 nm wavelength.

We have also investigated whether adjacent AuNPs that could form AuNP dimers within the gap region of the SiNW dimers and tetramers contribute to the SERS signal. Within the average 40 and 60 nm gaps of the SiNW dimers and SiNW tetramers, a AuNP–AuNP gap length of ca. 6 nm (SiNW dimer) and ca. 26 nm (SiNW tetramer) is to be expected, respectively. According to our FDTD simulations (785 nm excitation), compared to an isolated AuNP, these AuNP dimers provide a further increase in E-field strength by ca. 35% within a SiNW dimer, and ca. 6% within a SiNW tetramer (Figure 5). These results show that an even higher Raman signal enhancement is possible on the SiNW dimer sample when the AuNPs can directly couple to each other. Additionally, such AuNP dimers form extended hot spots where the E-field is significantly enhanced within the entire gap region, which might be beneficial for detecting large molecules via SERS or for performing “long-distance” SERS (Figure 5b). At 785 nm, the presence of AuNP dimers does not affect the overall E-field enhancement trend, e.g., dimers > tetramers > monomers. Similarly, E-field maps performed at 532 nm show that AuNP dimers located within the gap region of a SiNW dimer should also increase further the maximum E-field enhancement (Figure S6). This was not captured by our SERS measurements performed at 532 nm, which we attribute to the overall much lower E-field enhancement properties of the SiNW oligomers themselves (i.e., dimers and tetramers without AuNPs) at 532 nm compared to 785 nm (see Figure S5). Overall, while our FDTD results show that AuNP dimers forming within the gap region of SiNW dimers should positively contribute to the overall E-field enhancement, our experimental results suggest that the large Raman signal measured at 785 nm on all AuNPs@SiNW oligomer arrays can be predominantly attributed to the field enhancing properties of the SiNW oligomer arrays at this spectral region.

The remaining discrepancy between the FDTD simulation results and the Raman experiments most likely arises from (i) the geometrical inhomogeneity of the samples prepared in this work (not all nanowires are arranged in dimers and tetramers, respectively), (ii) the fact that slightly less efficient binding of the AuNPs into the tetramer central gap cannot be ruled out, and (iii) the random polarization direction of the incident E-field with respect to the dimer and tetramer long axis, which should decrease the average E-field enhancement provided by these samples compared to the SiNW monomer array.

Additionally, our FDTD simulations confirm that smaller gap sizes should provide larger E-field enhancements in the gap region (Figure S7), which could explain the larger Raman signal provided by the dimer sample (ca. 40 nm) compared to that of the tetramer sample (ca. 60 nm gap). However, even with a 40 nm gap size, the maximum E-field enhancement of the tetramer is still lower than that of the dimer structure (Figure S8). Thus, the difference in gap size cannot, by itself, explain the larger Raman signal measured on the dimer sample. Overall, our simulation and SERS results confirm the superior E-field enhancing properties of the dimer geometry at 785 nm and suggest that further increase in SERS effect could be obtained using dimers with smaller gaps.

## Conclusions

We report the synthesis, optical characterization, and SERS performance of SiNW monomer, dimer, and tetramer arrays prepared via a wet chemical approach combining colloidal lithography and MACE. After functionalization with an aminosilane, the SiNW oligomer arrays are coated with a dense monolayer of AuNPs. Remarkably, the AuNPs are able to enter the gap region between the SiNWs, which is, on average, only twice as large as the AuNPs themselves. SERS measurements revealed an approximately 3-fold increase in Raman signal of the AuNPs@SiNW dimer arrays, whereas the AuNPs@SiNW tetramers resulted in a ca. 2-fold increase compared to the AuNPs@SiNW monomer arrays. FDTD simulations and SERS experiments confirm the electromagnetic nature of the SERS effect observed at 785 nm on these substrates, where the largest E-field enhancements are likely to occur at the Au–Si interface within the gap region of the SiNW dimers. Owing to their high surface area, high E-field enhancing properties, and strong interaction with light, these AuNPs@VA-SiNW dimer arrays are interesting candidate structures to enhance capacities for sensing and photocatalytic applications.

## Experimental Section

### Materials

Unless stated otherwise, all of the chemicals mentioned here were used without further processing. APTES, 4-MBA, HAuCl_4_·3H_2_O, iodine, potassium iodide, and anhydrous toluene (99.8%) were acquired from Sigma-Aldrich. Acetone (99%), calcium chloride, ethanol (96%), isopropyl alcohol (≥98%), absolute ethanol (99.96%), and hydrofluoric acid (40%) were purchased from VWR. Hydrogen peroxide (30%) and trisodium citrate dihydrate were supplied by Merck. Sulfuric acid (95–97%) was obtained from Supelco. Milli-Q water used was double-deionized using a Milli-Q system with a resistivity of 18 MΩ. N-doped Si wafers (⟨100⟩, resistivity 1–30 Ωcm) were obtained from Si Materials, Germany. For the synthesis of SiO_2_@PDMAEMA core–shell particles used as a colloidal template, ethanol (EtOH; 99.9%, Merck), (*p*-chloromethyl)phenyltrimethoxysilane (95%, Gelest, Inc.), dimethylformamide (DMF, anhydrous, 99.8%, Sigma-Aldrich), tetraethyl orthosilicate (TEOS; 98%, Sigma-Aldrich), and ammonium hydroxide solution (28–30% NH_3_ basis, Sigma-Aldrich) were used as received. (2-Dimethylaminoethyl) methacrylate (DMAEMA; 99%, Merck) was passed over neutral aluminum oxide (Al_2_O_3_, Carl Roth) to remove the inhibitor. Sodium diethyldithiocarbamate trihydrate (Sigma-Aldrich) was recrystallized from methanol (99.8%, Sigma-Aldrich). Tetrahydrofuran (THF; 99.9%, Sigma-Aldrich) was dried by storage over activated molecular sieves. Water (H_2_O) was double-deionized using a Milli-Q system (18.2 M.cm, Elga PURELAB Flex).^56^

### Synthesis of the SiO_2_@PDMAEMA Core–Shell Particles

The detailed procedure was described elsewhere.^53^ In short the silicon dioxide (SiO_2_) cores were synthesized according to the Stöber method and functionalized with the iniferter immediately after the synthesis.^56,60^ 12.5 mL portion of H_2_O, 250 mL of EtOH, and 25 mL of ammonium hydroxide solution were mixed in a 500 mL round-bottom flask and heated to 50 °C in an oil bath. Under stirring at 1100 rpm, a preheated solution of 18.75 mL of TEOS in 75 mL of EtOH was added. After a reaction time of 18 h, 150 mL of the Stöber dispersion was removed and purified. 100 mg of the photoiniferter *N*,*N*-(diethylamino) dithiocarbamoylbenzyl-(trimethoxy)silane (SBDC), which was synthesized according to an established protocol,^53,61^ in 10 mL of EtOH was added to the remaining 231 mL of the Stöber dispersion and the dispersion was diluted with 170 mL of DMF. After another 24 h the functionalized cores were purified by centrifugation and redispersion four times in EtOH and four times in dry DMF. The yield was 2.6 g functionalized silica particles with a diameter of (170 ± 7) nm, as determined from electron microscopy images. For the synthesis of the core–shell particles, a dispersion of 0.9 g of functionalized cores in 162 mL of dry DMF was mixed with 18 mL of DMAEMA in a 250 mL round-bottom flask. The dispersion was deoxygenated by ultrasonication and flushing with argon four times. Then, the dispersion was placed in a UV-cross-linker (Vilber Bio-Link 365) and irradiated with 365 nm UV irradiation. After nine time intervals, 20 mL of the dispersion were removed from the reaction. The sample used during this study was irradiated for 160 min. The dispersion was purified by centrifugation and redispersion 10 times in EtOH, yielding 170 mg of core–shell particles with a hydrodynamic diameter of (481 ± 75) nm.

### Preparation of SiNW Monomer, SiNW Dimer, and SiNW Tetramer Arrays

A 1:1 mixture of absolute EtOH and the core–shell NP suspension was spread to the interface and deposited to the substrate at a 90° angle to the air–water interface. The Si was precleaned with ethanol and a 5 min oxygen plasma treatment (100 W, 4 sccm, Femto SLS, Diener, Germany) in order to ensure hydrophilicity of the substrate.^49,56^ Particle dimers and tetramers were assembled by continuously compressing the non-cross-linked core–shell particles to surface pressures of approximately 24 and 27 mN/m, respectively.^53^ For the subsequent step of MACE, the polymer shell of the core–shell NPs was completely removed during a 12 min treatment in oxygen plasma at 50 W. Then, a thin adhesion layer of aluminum-doped zinc oxide (AZO) was sputtered for 1 s at 75 W in a Clustex 100 M sputtering system by Leybold Optics. Additionally, Au was deposited using a Cressington Sputter Coater 108 auto (200 s at 40 mA) and the SiO_2_ spheres were lifted off using adhesive tape. The remaining Au nanohole film was used as an etching mask for the MACE procedure. After cleaning in oxygen plasma for 5 min at 50 W, the samples were etched for 1 min and 40 s in the MACE solution (10 mL H_2_O, 10 mL HF, and 0.75 mL H_2_O_2_) and rinsed three times in Milli-Q water followed by another 5 min etching step in diluted HF (20 mL H_2_O, 4 mL HF), which is necessary to remove residual porous SiO_2_ on the SiNW surface. Last, the samples were washed three times in Milli-Q water and once in ethanol before they were left dry in air.^30,31,39,50^

### Synthesis of AuNPs

For synthesizing AuNPs the citrate reduction process was employed.^57,62^ In particular, 25 mg of dry HAuCl_4_·3H_2_O was added to 250 mL Milli-Q water to prepare the Au stock solution. Subsequently, 90 mL of the Au stock solution was heated to boil under stirring in a 250 mL two-neck round-bottom flask under reflux. 5 mL of a 1 wt % solution of trisodium citrate in Milli-Q water was added to the boiling Au stock solution. After 5 min, stirring was stopped, but boiling of the solution was continued for another 25 min. Finally, an intense dark red color of the solution indicated the complete reduction of Au(III) ions and the formation of AuNPs. The as-prepared AuNP solution was filtered with a qualitative Whatman filter paper grade 1 before incubation with the SiNW arrays, as done previously.^7^

### Silane Functionalization and Assembly of AuNPs

In order to bind AuNPs to the surface of the SiNW arrays it was functionalized with (3-aminopropyl)triethoxysilane (APTES) as established previously in our group.^7^ First, the Au nanohole film from the MACE procedure at the bottom of the SiNWs was dissolved in an aqueous solution of KI/I_2_ (10 g KI and 5 g I_2_ dissolved in 85 g Milli-Q water) for at least 80 min followed by two washing steps in Milli-Q water and one washing step in ethanol. After a short oxygen plasma etching step (10 min at 50 W), SiNW samples were treated in piranha solution (Caution: Piranha solution is a 1:3 mixture of 30% hydrogen peroxide H_2_O_2_ and sulfuric acid H_2_SO_4_ and thus, extremely corrosive and strongly oxidizing for organics; risk of explosion if not handled with care)^63^ for 30 min and thoroughly rinsed in Milli-Q water. Directly afterward, an oxygen plasma treatment for 30 min at 100 W ensured the hydroxylation of the Si surface. After this step, samples can be stored in Milli-Q water.^7,64^

Next, SiNW samples were dried in a vacuum furnace at room temperature and transferred to a round-bottom flask with 50 mL of anhydrous toluene and 584 μL of APTES, corresponding to a 50 mM solution of APTES. After refluxing for 5 h at 90 °C using an oil bath, the flask was removed from the oil bath and cooled down still under reflux for at least 1 h and afterward closed. The next day, SiNW samples were transferred to 20 mL of fresh anhydrous toluene for 1 h and afterward dried in air. Directly after drying, the SiNW samples were annealed in a preheated oven at 90 °C for 2 h.^64,65^

Finally, the functionalized SiNW samples were rinsed with ethanol and washed for 1 h in Milli-Q water. This procedure was repeated a second time before the samples were left dried in air and then immediately transferred to 10 mL of a 1:1 mixture of absolute ethanol and AuNP solution (treated by ultrasound for 10 min directly before mixing). After 3 days of incubation, the SiNW samples were removed from the discolored AuNP solution, and rinsed with Milli-Q water twice.^7^

### Coupled SEM-Microscopy Diffuse Reflectance UV–vis Spectra

SEM images were obtained on a Zeiss GeminiSEM 500 (Carl Zeiss AG, Germany) at an operating voltage of 5 kV using the InLens Secondary Electron detector. The bright-field UV–vis reflectance spectra were recorded using a Zeiss Axio Imager Z2 light microscope with a 100× objective (numerical aperture NA = 0.75, EC Epiplan-NEOLFLUAR, Zeiss). An MCS CCD UV-NIR Spectrometer (Zeiss, Germany) was coupled to a light microscope in order to measure the reflectance spectra. The integration time per spectrum was set to 3500 ms. Further, the collected signal from the sample was limited to a 2 × 2 μm^2^ square by a mechanical aperture in the optical path toward the detector. A silver mirror (Thorlabs) was used as a reference for the bright-field spectra.

### Ensemble Reflectance Measurements

(Figure 2h) were performed using a PerkinElmer Lambda 1050 equipped with a 150 mm integration sphere. A white Spectralon reference was used for the baseline correction. During sample measurement, a black reference was placed behind the sample to avoid the influence of light reflection at the back of the samples. A circular 3 mm pinhole was used with a light focusing lens and a circular aperture to adjust the size of the light beam.

### SEM Images

A Zeiss Ultra Plus 55 instrument equipped with a field emission gun and Gemini lenses was used to acquire the shown SEM images. For topographical images, the InLens secondary electron (SE) detector and, for compositional contrast, the angle-selective backscattered electron (AsB) detector were used. For some of the SEM images shown in this work, the signals of both detectors were mixed.

SEM images were analyzed to determine the length, diameter, pitch, and gap sizes of the SiNW arrays using the freely available software ImageJ.^66^ The mean values for diameter and length were ca. 150 and 700 nm, respectively. The pitches of SiNW monomer arrays, SiNW dimer arrays, and SiNW tetramer arrays slightly varied and were determined to be ca. 550, 650, and 850 nm, respectively.

### Raman Measurements

The Raman spectra were obtained with a dispersive Thermo DXR2 Raman microscope (Thermo, USA) equipped with a confocal microscope BX41 by Olympus Corp. (Japan). Prior to the Raman experiment, three pieces of ca. 5 × 3 mm^2^ were cut off the cm-sized gradient sample, which were identified via SEM as homogeneous regions composed of SiNW monomers, dimers, and tetramers, respectively. After a short oxygen plasma treatment (1 min at 20 W) a 2 μL droplet of a 1 mM solution of 4-mercaptobenzoic acid (4-MBA) in isopropyl alcohol was drop-cast and then allowed to dry on the sample. One μL of a 1 mM solution of Rhodamine 6G (R6G) in ethanol and 1 μL of a 1 mM solution of malachite green (MG) in ethanol were dropped onto ca. 1 × 2 mm^2^ small pieces. Raman spectra were recorded with depolarized laser light at an excitation wavelength of 785 nm, a laser power of 10 mW (30 mW for the samples with R6G), and a 10× objective producing a laser spot with a diameter of 3.1 μm. In the confocal microscope setup, the 50 μm pinhole entrance was chosen, resulting in a spectral resolution of 4.7–8.7 cm^–1^ over the recorded spectral range between 200 and 3300 cm^–1^. An exposure time of 2 s and 3 accumulations per spectrum were chosen. In this work, the baselined mean of 25 spectra on 25 different positions of the samples is shown (Figures 3, S2, and S3). The autofocus option of the Raman spectrometer was employed before every measurement in order to optimize the laser focal point on the sample, and thereby achieve the largest Raman signal. Measurements at 532 nm were performed using a laser power of 5 mW while keeping all other measurement conditions equal.

### Electromagnetic FDTD Simulations

Electromagnetic simulations were carried out using the software Ansys Lumerical FDTD by Ansys Inc. (Canonsburg, USA).^55,67^ In order to simulate the optical response of Si, SiO_2_, and Au, the respective dielectric functions of those materials were used as they are available in the software. Periodic boundary conditions were used in *x* and *y* directions and perfectly matched layers (PML) boundary conditions in the *z* direction. All structures were simulated in a vacuum (*n* = 1). On top of the SiNWs, a 2 nm thin SiO_2_ shell was added in order to account for the oxide layer that is naturally growing on top of Si surfaces in air. The SiNW height was set to 700 nm and the SiNW diameter to 150 nm. A pitch of 550, 650, and 850 nm was used for the SiNW monomers, the SiNW dimers, and the SiNW tetramers, respectively, according to the values measured on the SEM images of the respective samples. A plane wave source emitting linearly polarized light was used to illuminate the simulated structures with light between 780 and 790 nm or 527 and 537 nm, respectively. Frequency-domain field profile monitors were used to investigate the E-field distributions on the simulated structures. A mesh size of 0.25 nm (2D) was used around the AuNPs and a mesh size of 4 nm (2D) was used around the SiNWs. In order to integrate the *E*^4^ around the SiNW, a 5 nm shell with a refractive index of 1.00001 was added around the SiNWs. The script of the advanced power analysis group by Lumerical was adjusted to derive and integrate the (E-field)^4^ components over the volume of the 5 nm shell.^7,54^

## Supporting Information Available

SEM images of different sample regions; Raman spectra of 4-MBA on the different substrates at 532 nm; Raman spectra of MG and R6G on the different substrates at 785 nm; scheme showing the orientation of the field monitors and definition of gap sizes used for the FDTD simulations; additional E-field maps of the dimers and tetramers (influence of the gap length); and additional E-field maps at 532 nm (PDF)

## Abbreviations

2D: two-dimensional or two-dimensional
3D: three-dimensional or three-dimensional
4-MBA: 4-mercaptobenzoic acid
APTES: (3-aminopropyl)triethoxysilane
AsB: angle-selective backscattered electron
Au: gold
AuNP: gold nanoparticle
AZO: aluminum-doped zinc oxide
CMOS: complementary metal-oxide-semiconductor
DMF: dimethylformamide
(D)RIE: (deep) reactive-ion etching
E-field: electric field
EtOH: ethanol
FDTD: finite-difference time-domain
IPA: isopropanol
LSP(R): localized surface plasmon (resonance)
MACE: metal-assisted chemical etching
MG: malachite green
NA: numerical aperture
NP: nanoparticle
NW: nanowire
PDMAEMA: poly(2-(dimethylamino)ethyl methacrylate)
R6G: Rhodamine 6G
RSD: relative standard deviation
SE: secondary electron
SERS: surface-enhanced Raman spectroscopy
SEM: scanning electron microscopy
Si: silicon
SiNW: silicon nanowire
TEOS: tetraethyl orthosilicate
UV–vis: ultraviolet–visible
VA-SiNW: vertically aligned silicon nanowire
VLS: vapor–liquid–solid
