# Supplementary material for: Au Nanoparticles@Si Nanowire Oligomer Arrays for SERS: Dimers Are Best
Source: ACS Appl Mater Interfaces. 2024 Jul 26;16(31):41379–89. doi: 10.1021/acsami.4c10004 (PMC11310913; doi:10.1021/acsami.4c10004)
Supplement: Supplementary file 1 — am4c10004_si_001.pdf [file am4c10004_si_001.pdf]

Supporting Information

Au Nanoparticles@Si Nanowire Oligomer Arrays for SERS: Dimers are Best

*Theresa Bartschmid,[1] Johannes Menath,[2] Lukas Roemling,[2] Nicolas Vogel,[2] Furkan Atalay,[1] Amin Farhadi,[1] Gilles R. Bourret[1,]**

[1] Department of Chemistry and Physics of Materials, University of Salzburg, Jakob Haringer Strasse 2A, A-5020 Salzburg, Austria

[2] Institute of Particle Technology, Friedrich-Alexander University Erlangen-Nürnberg, Cauerstrasse 4, 91058 Erlangen, Germany

*E-mail: gilles.bourret@plus.ac.at

Supporting InformationFigures.

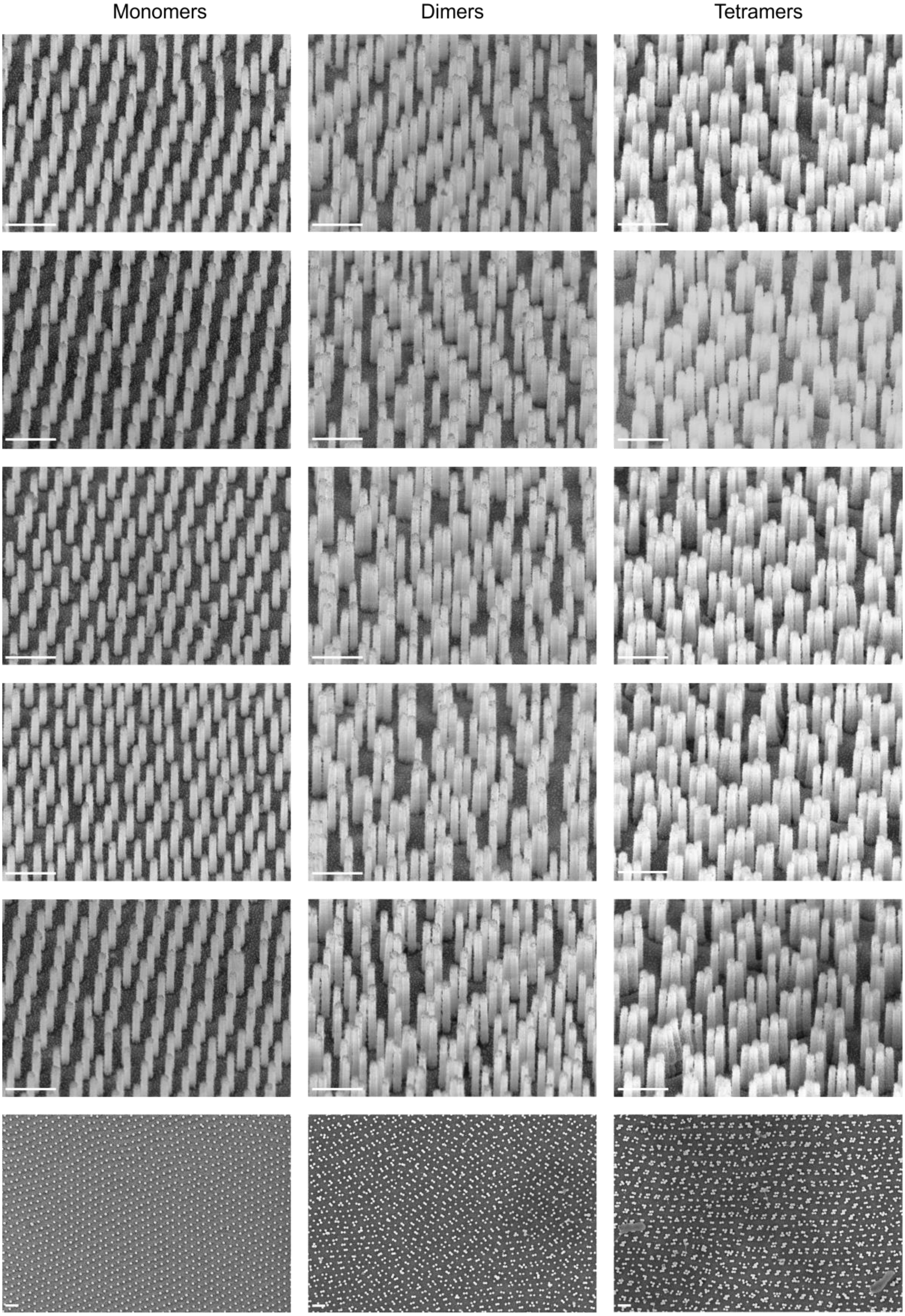

Figure S1. SEM images of SiNW monomer (left column), SiNW dimer (center column) and SiNW tetramer (right column) arrays with AuNPs showing the homogeneity of the SiNW structures as well as the coverage with AuNPs. Tilted view (45°) (top rows) and top view (bottom row) SEM images. Scale bars: 1 µm.

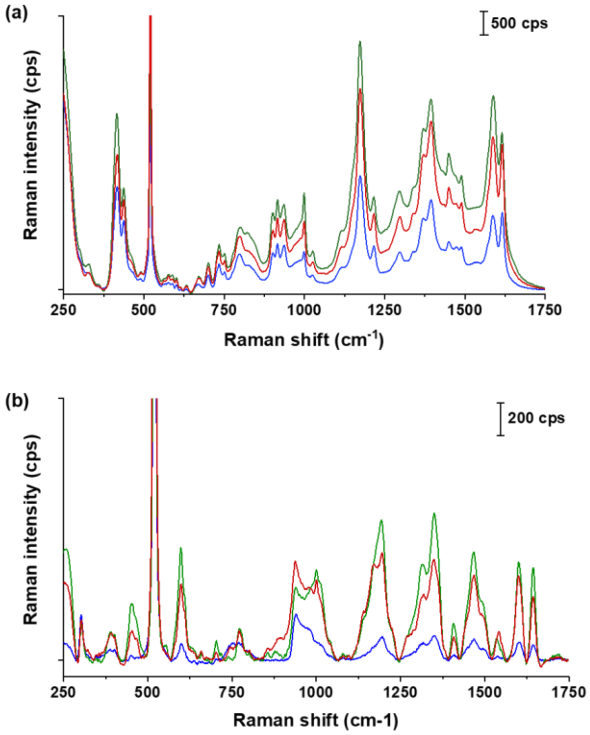

Figure S2. Baseline corrected Raman spectra recorded with 785 nm laser excitation on the SiNW monomer arrays with AuNPs (blue), on the SiNW dimer arrays with AuNPs (green) and on the SiNW tetramer arrays with AuNPs (red) utilizing the analyte molecules malachite green (a) and Rhodamine 6G (b).

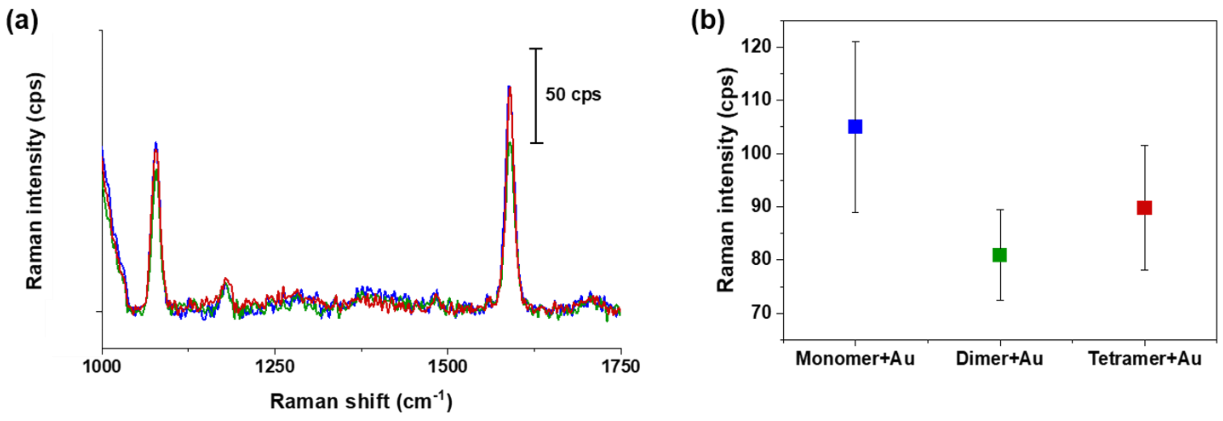

Figure S3. (a) Baseline corrected Raman spectra recorded with 532 nm laser excitation on the SiNW monomer arrays with AuNPs (blue), on the SiNW dimer arrays with AuNPs (green) and on the SiNW tetramer arrays with AuNPs (red). (b) Respective mean values and standard deviations for the peak of the analyte 4-MBA at 1075 cm^{-1}.[1]

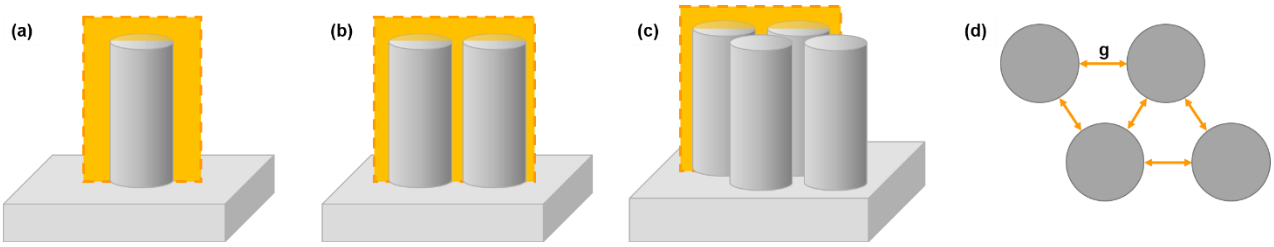

Figure S4. Scheme showing the location of E-field monitors (orange) used in the FDTD simulations to obtain the E-field enhancement maps of the SiNW monomer (a), SiNW dimer and SiNW tetramer (c) arrays. (d) Scheme showing the distances measured to obtain the average gap size of the SiNW tetramers, which were then used for the FDTD simulations.

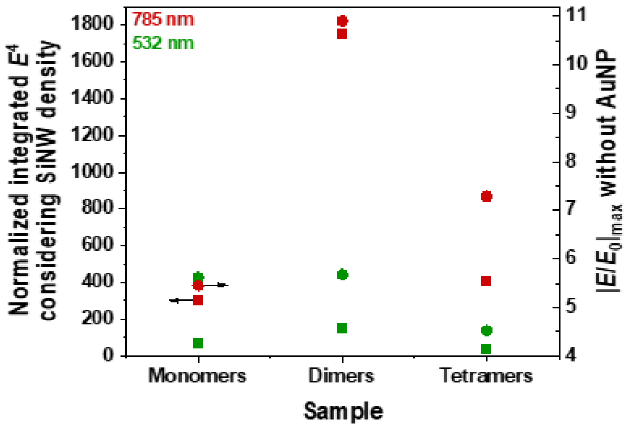

Figure S5. Normalized integrated E^4 over a 5 nm shell around the SiNWs normalized by the integration volume considering the SiNW density (square symbols; left axis) and maximum enhancement of the E-field strength (circular symbols; right axis) on SiNW monomer, dimer and tetramer arrays without AuNPs at 785 nm (red) and 532 nm (green).

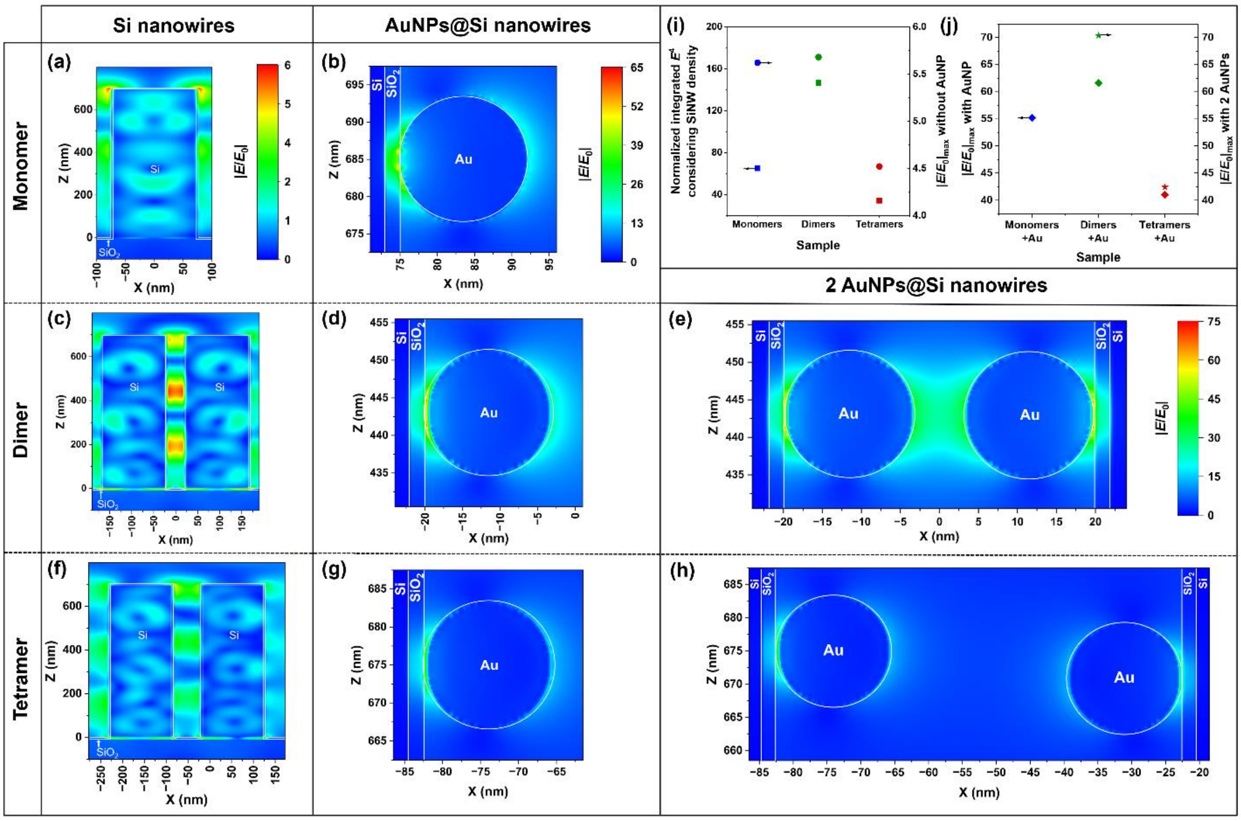

Figure S6. (a, c, f) Simulated E-field enhancement maps of a SiNW monomer (a), dimer with a 40 nm gap (c), and tetramer with a 60 nm gap (f), same color scale, shown in (a). Corresponding E-field maps with a AuNP (17 nm diameter) located in a region of increased E-field for the SiNW monomer (b), dimer (d) and tetramer (g), using the same color scale, shown in (b). Corresponding E-field maps with 2 AuNPs (17 nm diameter) located in a region of increased E-field for the dimer (e) and tetramer (h), using the same color scale, shown in (e). A native 2 nm-thick SiO_2 layer is used in all the simulations. (i) E^4 integrated over a 5 nm shell around the SiNW geometry and normalized by the integration volume and multiplied by the SiNW density (square symbols, left axis), and maximum enhancement of the E-field strength without AuNP (full circle symbols, right axis). (j) Maximum enhancement of the E-field strength with a AuNP located in the region of increased field intensity for the three different SiNW samples (diamond symbols, left) and with 2 AuNPs located in the region of increased field intensity for the dimer and the tetramer (star symbols, right). Light was injected along the Z axis and polarized along the X axis with a 532 nm excitation wavelength.

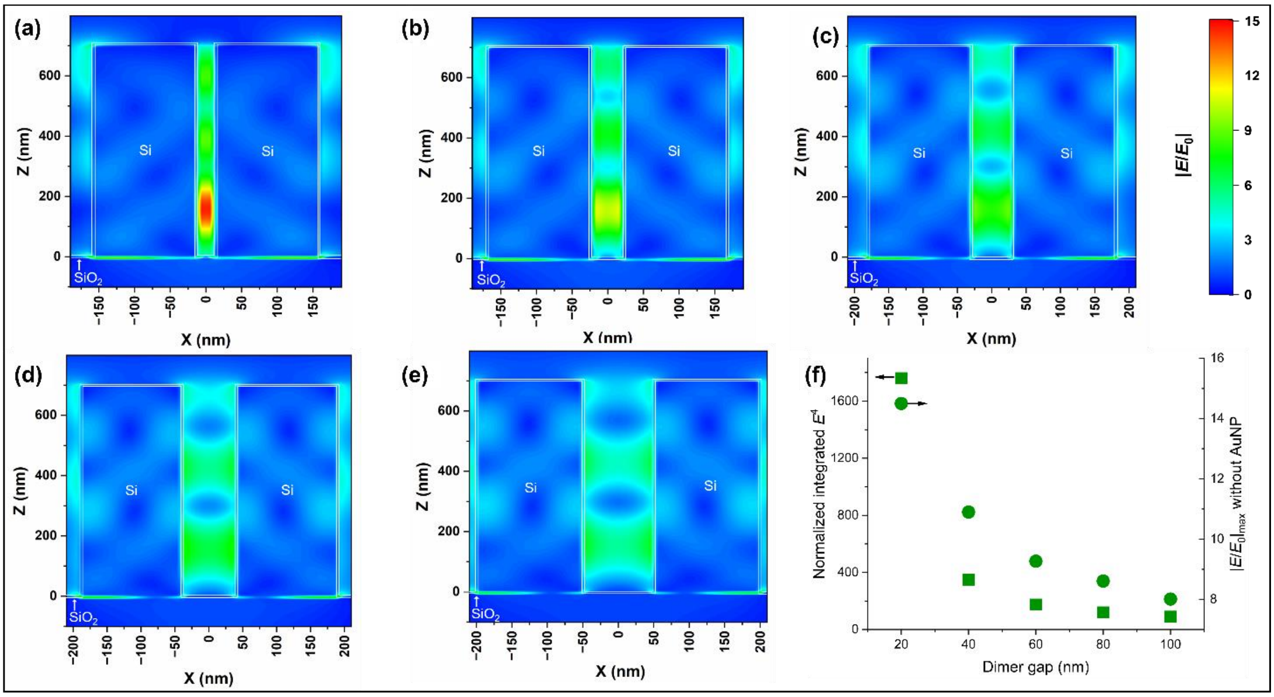

Figure S7. Electromagnetic FDTD simulations of E-field maps of SiNW dimer arrays with gap sizes of 20 nm (a), 40 nm (b), 60 nm (c), 80 nm (d) and 100 nm (e). (f) Integrated E^4 over a 5 nm shell around the SiNWs normalized by the integration volume (square symbol, left axis) and total maximum E-field enhancement (full circle symbol, right axis). Light was injected along the Z axis and polarized along the X axis and with a wavelength of 785 nm.

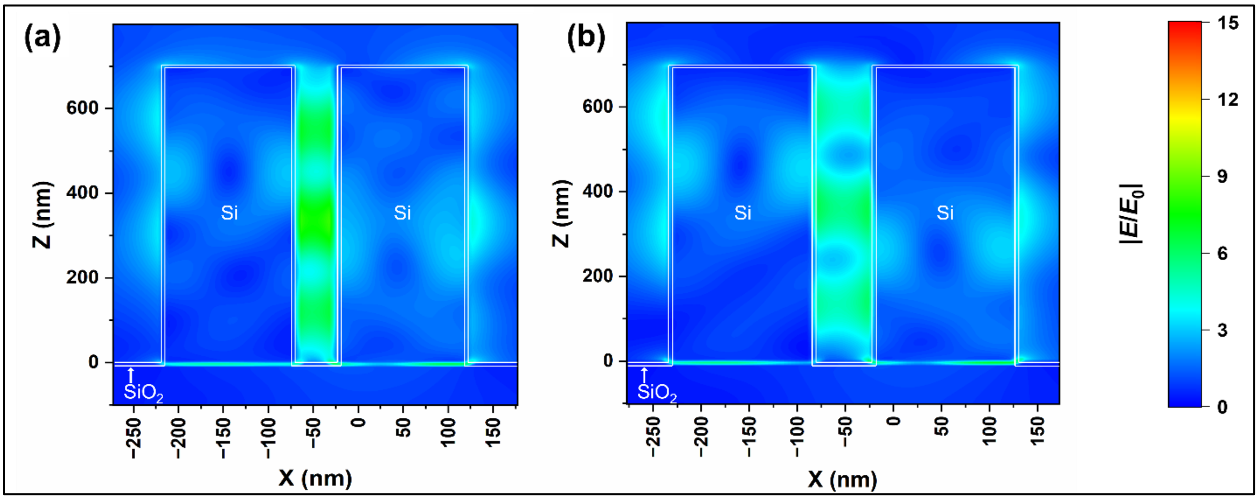

Figure S8. Electromagnetic FDTD simulations of E-field maps of SiNW tetramer arrays with gap sizes of 40 nm (a, $|E/E_0|_{max}$ = 8.4) and 60 nm (b, $|E/E_0|_{max}$ = 7.3). Light was injected along the Z axis and polarized along the X axis and with a wavelength of 785 nm.

References.

1. Michota, A.; Bukowska, J. Surface-enhanced Raman scattering (SERS) of 4-mercaptobenzoic acid on silver and gold substrates. *J. Raman Spectrosc.* **2003**, *34* (1), 21-25.
